# Cases

**Published:** 1885-08

**Authors:** J. W. Thomson


### CLINICAL BUREAU.

### CASES.

Mr. G. B., æt. fifty-one. September 23d, 1884.—Since 1st of last June rheumatism of left shoulder and arm. Sat in car by open window while wet; following week pain and stiffness in left shoulder and arm; now can only lift arm to head by elevating it with right hand; at biceps feels as though had been struck with a club and paralyzed; for about three months has not slept two hours consecutively; always awakened by intense shooting pain in arm caused by turning on left side; the arm felt as though it had received a blow—thus, has to lie on right side or back; cannot lie on painful side; does not get refreshing sleep, and feels that the trouble is wearing on his health; thirst for large quantities all the time; drinks over two quarts per diem; micturition frequent and profuse; cannot hold urine any length of time; pulse very full—eighty-six.

His old-school physician has frequently used the galvanic battery and given him medicine, but without relief. He has also taken mineral waters with a similar result.

Two years ago had severe chill, followed by fever and sweat, with thirst all through chill, fever, and sweat.

One dose Acon.$^{54m}$.

October 3d.—Did not feel slightest change from action of remedy till ninth day, then, for the first time for months, slept all night —from 12 to 6.30 A. M.—without awakening. When awoke, was lying on left side, refreshed and invigorated. The restoration came unconsciously during sleep. Slept as well on succeeding night. He took pleasure in showing that he could now lift arm to head or throw it backward. In the night while he slept the fruition came.

No improvement as yet in the abnormal thirst or micturition. No more medicine.

October 10th.—Less thirst, and does not urinate so much or so often. Sudden change in weather had caused severe pain in left arm. Placebo.

October 16th.—Pain from shoulder to elbow continues and it feels stiff. Acon.$^{cm}$, one dose.

October 28th.—Said he was entirely better, but walked four miles in a political procession, which overtaxed him, and he had taken cold. Acon.$^{200}$ every two hours till three doses were taken.

He is well, and has had no trouble since with his physical man.

J. W. THOMSON.

---

I would like to call attention to a symptom which I have *cured* several times with Bell. "high," and one which I have learned to regard as a "key-note," especially when prescribing for acute diseases—quinsy, diphtheria, etc.—viz.: *a sensation as though bubbles were bursting in the ears, especially the right one.*

JOHNSTONE.

